# Acceptance and Utilisation of Sulphadoxine-Pyrimethamine and Insecticide-Treated Nets among Pregnant Women in Oyo State, Nigeria

**DOI:** 10.1155/2015/713987

**Published:** 2015-12-29

**Authors:** Aderonke A. Adeola, Eugenia A. Okwilagwe

**Affiliations:** ^1^Federal Training Centre for Teachers of Health Sciences, University College Hospital, Ibadan 02234, Nigeria; ^2^Institute of Education, University of Ibadan, Ibadan, Nigeria

## Abstract

The study is an investigation of the acceptance and utilisation of Sulphadoxine-Pyrimethamine (Fansidar), the drug of choice for Intermittent Preventive Treatment in pregnancy, and Insecticide-Treated Nets among pregnant women who access different health facilities in Oyo State, Nigeria. Pregnant women (582) attending government primary healthcare antenatal clinics and 50 attending faith clinics purposively selected responded to structured instruments that were analysed using percentages, *t*-test correlation, and multiple regression. Acceptance and utilisation of RBM tools were higher in government clinics than faith clinics and in rural areas. Pregnant women in government clinics, 60.8% and 66.8%, and faith clinics, 18% and 38.0%, utilised Roll Back Malaria tools, significant at *t*_(630)_ = 5.81, *p* ≤ 0.05, and *t*_(630)_ = 3.99, *p* ≤ 0.05, respectively. Pregnant women in rural locations who accessed government clinics utilised Roll Back Malaria tools more than those in urban areas, *t*_(580)_ = −641, *p* ≤ 0.05. Number of pregnancies, educational qualification of the pregnant women, and marital status significantly and consistently influenced acceptance and utilisation of these tools. To ensure that set targets are met, the utilization of RBM tools among the two categories of pregnant women can be improved by increasing the supply of the tools and ensuring that treatment is free.

## 1. Introduction

Malaria has been man's problem since the 14th century (the era of the black death in Europe), and now in the 21st century, its effect has again reached an alarming proportion. Malaria is the major and commonest cause of ill-health in Africa. It affects up to 500 million people worldwide annually [1]. Malaria remains a major threat to health and economic development of local communities and nations [2, 3]. Almost half of the world's population is at risk of the disease [4–6]. Africa South of the Sahara records about 90% of the world's all malaria deaths [7] for two main reasons. The first is that infections are caused by* Plasmodium falciparum* (*P. f.*) (the most dangerous of the four human malaria parasites) and the second is because the mosquito* Anopheles gambiae* (the most effective malaria vector and also the most difficult to control) is most widespread in Africa [7].

Malaria infection during pregnancy is “a major public health problem in tropical and sub-tropical regions throughout the world” [8]. Children under 5 of years of age and pregnant women are recognised by the World Health Organization [9] as the vulnerable groups to malaria infection. The disease causes about 300–500 million clinical cases and 1.5 to 2.7 million deaths worldwide each year (WHO in [10]) even though there is a downward trend in these figures. The prevalence of malaria in Africa is high with an estimated average prevalence of* P. f.* of 63% in West Africa and 39% in Eastern and Southern Africa. Reports of outpatient visits due to malaria for some Africa countries such as Malawi, UR Tanzania, Uganda, and Zambia vary from 18% in 1985 to 46% in 2000 while hospital admission due to malaria in these countries varies from 20% in 1985 to 60% in 2000 according to RBM [7]. Child mortality due to malaria seems to have been higher in Eastern/Southern Africa countries than in West African countries between 1990 and 1998. Malaria accounts for “63% of the diseases reported in healthcare facilities across the six geopolitical zones of Nigeria,” while it accounts for “11% of maternal deaths” and its prevalence among pregnant women was put at “47% malaria situation” in Nigeria in [4].

Insecticide-Treated Nets (ITNs) are known to be highly effective in the reduction of morbidity and mortality in malaria RBM [11]. In recent times, their usage in the African continent has been vigorously scaled up for coverage and in the introduction of Long-Lasting Insecticide Nets (LLINs). ITNs have been scaled up in many African countries among which are Tanzania, Zambia, Kenya, Mali, and Malawi (RBM [11]). In these countries, ITNs are usually distributed freely to parents of children and pregnant women (the vulnerable groups) while, in some countries, Nigeria inclusive, they are made available at retail shops and/or are supplied at subsidised prices or under the voucher scheme [12, 13].

RBM programme is put in place to assist endemic countries to conduct national needs assessments from which strategic plans could be developed. It also intended to serve as a forum to match country's plans with international donors. In view of the complications of malaria in pregnancy, especially in this period of drug resistant malaria parasites, Roll Back Malaria (RBM) programme was launched in 1998 with at least three new or refined tools to combat the disease [9]. The tools are “Artemisinin-Based Combination Therapies” (ACTs), “Insecticide-Treated Nets” (ITNs), and “Intermittent Preventive Treatment” (IPT) for pregnant women. Besides, other RBM measures used for malaria control are IRS, larval control, quick diagnosis, administration of Artemisinin-Based Combination Therapies (ACTs) (freely given in some countries for all age groups), and adoption of chloroquine, mefloquine, and Sulphadoxine-Pyrimethamine (SP) (Fansidar) and primaquine. In spite of the efforts galvanised to combat malaria in Africa, there is still a high burden of malaria of 20%–40% with an average of 30% of all outpatient visits to the clinics in all African countries where malaria is endemic (RBM [7]). In many of these countries, the report indicated that between 20% and 50% of all hospital admissions are due to malaria. Reports (RBM [7]) indicate that less than 40% of malaria morbidity and mortality occur in formal health facilities which constitute a small fraction of the total burden but many of the reports do not include nongovernmental facilities like the faith clinics.

By 2005, it was expected that 60% of pregnant women in Nigeria would sleep under an ITN and 60% of pregnant women would receive IPT using Sulphadoxine-Pyrimethamine (SP) at least twice during ANC [4]. These targets were to gradually rise to a value between 75 and 80% by 2010 and coverage was sustained thereafter [12]. A national malaria situation survey conducted in 2000 reported by [4] indicated that preventive health behaviour in malaria in terms of net use among pregnant women in Nigeria seems to be generally low in all the six geopolitical zones. Also, [4] reported a generally poor usage of nets among all categories of people in Nigeria with only 20% of households owning nets, some of which are not necessarily ITNs, whereas [14] observed a higher coverage of the usage of nets in urban areas.

The need to further confirm current state of usage of RBM tools among this vulnerable group and the influence of location in this regard was crucial. In view of this, the present study had the impetus to investigate the acceptability and utilisation of RBM tools, their influencing factors, and the influence of location on the behaviour outcomes among pregnant women who access government and faith clinics in Oyo State, Nigeria, which seem to be usually sidelined by most health programmes initiated even when a sizeable number of pregnant women patronise them.

## 2. Research Questions

1. What is the level of acceptance of Intermittent Preventive Treatment (IPT) of malaria in pregnancy and ITNs by pregnant women who attend different health facilities in Oyo State?
2. Is there a difference in acceptance of IPT and ITNs by pregnant women in terms of the health facilities accessed and location?
3. To what extent are IPT and ITNs utilised by pregnant women who access different health facilities in Oyo State?
4. Is the utilisation of IPT and ITNs by pregnant women a function of the health facility accessed and location?
5. Is there a relationship between acceptance and utilisation of IPT and ITNs by these pregnant women?
6. What are the factors influencing (i) acceptance and (ii) utilisation of IPT and ITNs among pregnant women who access these health facilities in Oyo State?

## 3. Materials and Methods

### 3.1. Design

The study adopted the survey and causal comparative types of research as none of the variables were manipulated; they were studied as they occurred and inferences made on the basis of the findings obtained. The levels of acceptance and utilisation of Sulphadoxine-Pyrimethamine (Fansidar), the drug of choice for Intermittent Preventive Treatment (IPT) in pregnancy, and Insecticide-Treated Nets (ITNs) between government clinics and faith clinics or mission homes were compared.

### 3.2. Study Area and Sample

The study was conducted in Oyo State, Nigeria. Oyo State is located in the Southwest Geopolitical Zone of Nigeria and is one of the malaria endemic areas of the country. It consists of 33 local government areas with its capital city at Ibadan. The disease afflicts children under five and pregnant women—two of the most vulnerable groups—resulting in high morbidity and mortality in the state. For instance, data from [15] puts the trend of malaria prevalence in the state from a total of 358,780 cases in 2006 to 403,468 in 2009. The study population consisted of all pregnant women in Oyo State, Nigeria, who were attending antenatal (ANC) clinics at both the Primary Healthcare Centres (PHC) and faith clinics, respectively, in 2009-2010 when the study was conducted. The 33 local government areas in the state were stratified into urban and rural composition and purposive sampling technique was employed to select three local government areas, respectively, from both urban and rural locations to take care of the location of the faith clinics/mission homes which were dominant in urban locations. All the PHC clinics and mission homes (faith clinics) in the selected local government areas were used for the study, while purposive sampling technique was used to select a total of 650 pregnant women but 582 who accessed government clinics and 50 who accessed faith clinics in these LGAs who had complete information on them formed the final sample.

### 3.3. Instrumentation

A structured questionnaire that was valid and reliable—Pregnant Women Acceptance and Utilisation Questionnaire (PWAUQ, *α* = 0.81)—used for data collection was administered once to the participants. PWAUQ consisted of background information about the pregnant women and their spouses' occupations and educational levels, locations, and number of pregnancies and their outcomes among others. The instruments on acceptance of RBM tools, utilisation of ITNs, and factors influencing acceptance and utilisation were closed-ended questions administered once to the participants. Data collection was carried out by the researchers with the assistance of fourteen trained assistants (six RBM programme managers in the six LGAs and eight others trained for the purpose of data collection). The researchers explained the purpose of the research work to all pregnant women that were present including the health workers at each PHC or faith clinic or mission home visited. Pregnant women in rural locations who had low educational qualification filled their questionnaire through the assistants who interpreted the contents to them in the local language Yoruba without influencing their responses.

### 3.4. Data Analysis

The data obtained were coded and analysed using the Statistical Package for the Social Sciences (SPSS) Version 20. Analyses of valid items were carried out and nonrespondents were excluded. Positive responses were coded with one (1) while negative responses were coded with zero (0). Percentages were computed for the responses relating to levels of acceptance and utilisation of RBM tools. Inferential statistics such as *t*-test and Analysis of Variance (ANOVA) were computed to determine significant levels of acceptance and utilisation of RBM tools between pregnant women who accessed government and faith-based health facilities and those in urban and rural locations. *p* values of ≤ 0.05 were considered as an acceptable level of significance. Pearson product moment correction was used to analyse the relationship between acceptance and utilisation of RBM tools. Multiple regressions were computed to establish the factors that influenced the acceptance and utilisation of the RBM tools.

## 4. Results

1. What is the level of acceptance of Intermittent Preventive Treatment (IPT) of malaria in pregnancy and ITNs by pregnant women attending different health facilities in Oyo State?

Pregnant women who accessed government clinics (49.8%) tended to accept ITNs even though 72% of them claimed to “like sleeping under a mosquito net,” while 52.2% claimed they “accept IPT.” A total of 28% and 30% of pregnant women who accessed faith clinics reported that they accepted these RBM tools, respectively, though 72% of them indicated they “like sleeping under a mosquito net” and 64% “react to anti-malarial drugs” (Table 1).

(2) Is there a difference in the acceptance of IPT and ITNs by pregnant women in terms of the health facility accessed and location?

There was a significant difference in acceptance of IPT and ITNs by pregnant women in the two health facilities in Table 2, *t*_(630)_ = 3.51, *p* ≤ 0.05, and *t*_(630)_ = 4.15, *p* ≤ 0.05, respectively. Pregnant women in government clinics with M = 4.74, SD = 1.08 and M = 4.66, SD = 1.15 seem to accept IPT and ITNs compared to their counterparts in faith clinics, Mean = 4.16, SD = 1.59; M = 3.96, SD = 0.93. The effect sizes (Cohen's *d*) of 0.14 or 14% and 0.16 or 16%, respectively, were obtained for the variance in acceptance in the two health facilities.

Significant influence of location was observed on the extent to which pregnant women in government health facility only accepted IPT (Table 3). Pregnant women in rural locations with Mean 4.92, SD = 1.00 were better in the acceptance of IPT than those in the urban areas, Mean 4.55, SD = 1.13, *t*_(580)_ = −4.12, *p* ≤ 0.05, with an effect size (Cohen's *d*) of 0.17 or 17%. The influence of location on acceptance of ITNs was not significant, *t*_(580)_ = −0.58, *p* ≤ 0.05. Faith clinics were not found in rural locations.

(3) To what extent are IPT and ITNs utilised by pregnant women attending different health facilities in Oyo State?

Pregnant women who accessed government clinics (60.8%) utilised ITNs in spite of the claim that 62.9% reported “sleeping under an ITN before their current pregnancy” and 73.2% claimed “it is comfortable sleeping under an ITN.” Among pregnant women who accessed faith clinics, 18% utilise ITNs even though 48% claimed they “find sleeping under an ITN comfortable.” More women (66.8%) in government clinics than their counterparts in faith clinics (38.0%) used IPT to the expected two doses. As many as 79% of the pregnant women in government clinics and 62% in faith clinics indicated their willingness to use IPT in their subsequent pregnancies (Table 4).

(4) Is the utilisation of IPT and ITNs by pregnant women a function of the health facility accessed and location?

A significant difference in the utilisation of IPT and ITNs as presented in Table 5 was observed between these women, *t*_(630)_ = 5.81, *p* ≤ 0.05, and *t*_(630)_ = 3.99, *p* ≤ 0.05. Pregnant women in government clinics utilised IPT and ITNs more than those who accessed faith clinics. The effect sizes (Cohen's *d*) of 0.23 or 23% and 0.16 or 16%, respectively, were obtained for the variance in the utilisation of IPT and ITNs, respectively.

A significant difference in the utilisation of IPT by the pregnant women who accessed government clinics by location, *t*_(580)_ = −641, *p* ≤ 0.05, was observed with an effect size of 0.26 or 26% but the ITNs utilisation was not significant, *t*_(580)_ = 1.170, *p* > 0.05, with an effect size of 0.05 or 4.86% (Table 6). Faith clinics were not found in rural locations.

(5) Is there a relationship between acceptance and utilisation of RBM tools by these pregnant women?

As presented in Table 7, the utilisation of RBM tools which was found to be significantly related with the acceptance of RBM tools among pregnant women in the state government clinics (*r* = 0.398, *N* = 582,   *p* ≤ 0.05;  *r* ^2^ = 0.16) and pregnant women in faith clinics (*r* = 0.379, *p* ≤ 0.05; *r* ^2^ = 0.14). This explained 16% and 14% of the variation between acceptance and utilisation among these women, respectively.

(6) What are the factors influencing (i) acceptance and (ii) utilisation of IPT and ITNs among pregnant women who access these health facilities in Oyo State?

The multiple regression model summary of the nine influencing variables (Table 8) significantly explained and predicted the acceptance of RBM tools among pregnant women in government clinics (*F*_(9,572)_ = 6.320, *p* ≤ 0.05) but not in faith clinics. The variables accounted for 8% of variance in the pregnant women's acceptance of RBM tools whereas other variables not investigated accounted for the remaining 92%. With respect to the *t*-test results in relation to the significant multiple regression coefficient obtained, the nine variables significantly predicted the acceptance of RBM tools through number of pregnancies (*t* = 5.172, *β* = 0.217). Four other variables, educational qualification of the pregnant woman (*t* = −2.871, *β* = −0.120), marital status (*t* = −2.928, *β* = −0.117), age of pregnant woman (*t* = −2.805; *β* = −0.115), and husband's occupation (*t* = −0.2212, *β* = −0.093), also contributed significantly but inversely to the acceptance of RBM tools (Table 8).

The multiple regression of the nine influencing independent variables of the utilisation significantly explained and predicted the utilisation of RBM tools among pregnant women in government clinics (*F*_(9,572)_ = 3.607, *p* ≤ 0.05) but not in faith clinics (Table 9).

The nine variables accounted for 3.9% variance in the utilisation of RBM tool whereas other variables not investigated accounted for the remaining 96.1%. Concerning the *t*-test results in relation to the significant effect of the multiple regression coefficient obtained, the nine variables significantly predicted the utilisation of RBM tools by pregnant women in government clinics through the number of pregnancies had (*t* = 2.818, *β* = 0.121). Marital status and educational qualification of the pregnant women also contributed significantly but inversely to the utilisation of RBM tools among these women (*t* = −3.248, *β* = −0.132 and *t* = −2.496, *β* = −0.106, resp.).

## 5. Discussion

### 5.1. Acceptance and Utilisation of RBM Tools in Pregnancy

The findings with respect to the acceptance of RBM tools (IPT and ITNs) by pregnant women indicated that pregnant women who accessed government clinics accepted these RBM tools better than those who accessed faith clinics. Between 50% and 72% of the pregnant women who accessed government clinics accepted IPT and ITNs whereas between 30% and 72% of pregnant women who accessed faith clinics accepted these RBM tools. The overall acceptance by these women (even when pregnant women who accessed faith clinics claimed they engage in dual registration) could be as a result of adequate enlightenment on the knowledge of RBM programme provided to the pregnant women in government clinics through health education at ANC visits, which is a regular practice of primary healthcare in the clinics. This must have contributed to the favourable acceptance of the RBM tools. The targets of over 82% in the use of RBM tools by pregnant women were set by the African Heads of State to be achieved by year 2010. The observed trend in this study is an indication that these targets are yet to be fully achieved. The level of education of an individual generally dichotomises one into an ignorant or informed person. According to [16], health education helps people understand health and how their behaviour may affect their health. Since such education encourages people to make their own choices for a healthy life, it will ensure the success of the prevention, treatment, and control of malaria programme put in place in order to reduce to the barest minimum maternal and child morbidity and mortality, in the state.

Other findings in this study indicate that health facility accessed by the pregnant women significantly influenced acceptance: government clinics: *t*_(630)_ = 3.51, *p* ≤ 0.05; faith clinics: *t*_(630)_ = 4.15, *p* ≤ 0.05. Acceptance was also location based as women in the rural locations significantly accepted IPT in pregnancy compared to those in urban locations (*t* = 4.12, *p* ≤ 0.05) but location did not significantly influence the acceptance of ITNs for this group of women. This implied that RBM programme in Oyo State is a healthcare focused programme geared towards the rural people through the primary healthcare. This way, the study findings corroborated [17]. Thus, the rural dwellers see RBM programme as their own programme which they embrace wholeheartedly more than the urban dwellers. Personal experiences of the researchers show that dwellers in the urban areas take malaria for granted and probably see it as a common disease occurrence.

Findings indicate a modest level of the utilisation of IPT and ITNs by the pregnant women. More of the pregnant women in government clinics (between 58% and 73%) utilised IPT and 67.2% used ITNs whereas between 18% and 60% and 38.0% of those who accessed faith clinics used these tools, respectively. This was significant at *p* ≤ 0.05, respectively. These figures are slightly better than the 2005 targets. The observed difference could be attributed to the religious beliefs and practices of the women in faith clinics who do not believe in the use of drugs even though they engage in dual registration. The study of [13] finds support in respect of this finding. Although researchers like [17] revealed that factors such as availability of nets, cost of nets, and inadequate information on where to obtain the nets and the attitude of users towards the use of net had great influence on the poor and improper utilisation of nets (ITNs), the findings of this present study seem to have indicated a considerable improvement in the utilisation of ITNs among the pregnant women. Findings differed markedly from the findings of [18] that reported less than 10% usage of nets among pregnant women and children in Uganda but are in consonance with the [19] study which reported 58% usage of ITNs among women attending ANC and delivery units in Burkina Faso and [20] that reported a variation between 32% and 69% usage of ITNs among pregnant women in six African countries, Nigeria inclusive. Findings equally find support in the study of [21] which found that ITNs use in Niger improved after a nationwide integrated campaign.

The influence of location on the utilisation of RBM tools by the pregnant women in this study was important as pregnant women in rural locations who accessed government clinics utilised IPT compared to those in urban areas, significant at *p* ≤ 0.05. In this regard, findings in Nigeria [22, 23] which corroborated [14] are in support of the fact that the use of nets was very variable among urban and rural communities in Oyo and Ekiti States, respectively. A national malaria situation survey conducted in 2000 reported by [4] also indicated that net use among pregnant women in Nigeria seems to be generally low in all the six geopolitical zones. It also indicated a generally poor usage of nets among all categories of people in Nigeria with only 20% of households owning nets, some of which are not necessarily ITNs, whereas [14] observed a higher coverage of the usage of nets in urban areas. There is therefore need to further reach pregnant women in urban locations to improve their attitude to IPT and ITNs utilisation as well as those who access faith clinics.

In addition, a positive but low correlation seems to exist between acceptance and utilisation of IPT and ITNs among pregnant women who accessed government clinics (*r* = 0.398) accounting for 16% variance in utilisation compared to those who accessed faith clinics (*r* = 0.379) accounting for 14% variance in utilisation. This implied that, all things being equal, high acceptance implies reliable utilisation of IPT and other RBM programme packages. The acceptability of and receptiveness to RBM tools by some of the pregnant women could have facilitated their utilisation of the tools with ease. The implication of this finding is that any effective public enlightenment and sensitisation carried out on RBM programme and tools will make the right impact on reducing the menace of malaria on pregnant women irrespective of the health facility accessed.

### 5.2. Factors Influencing Acceptance and Utilisation of Roll Back Malaria Tools

From the findings in this study, pregnant women in the state have a high acceptance of RBM tools. It appears, however, that the acceptance of these tools by pregnant women who accessed government clinics depended on factors such as number of pregnancies had (*β* = 0.217) which significantly and positively influenced these women's acceptance of these tools, but educational qualification of the pregnant woman (*β* = −0.120), marital status (*β* = −0.117), age of the pregnant women (*β* = −0.115), and husband's occupation (*β* = −0.093), though significant, inversely influenced acceptance. Other factors were not significant. What these findings imply is that as the age increased or educational qualification and marital status or husbands' occupations of these women improved, their acceptance of RBM tools became low. On the issue of the utilisation of the tools by pregnant women who accessed government clinics, the findings revealed that utilisation was influenced by such factors as the number of pregnancies had (*β* = 0.121) which contributed significantly and positively to utilisation and marital status (*β* = −0.132) and educational qualification of the pregnant women (*β* = −0.106) which contributed significantly and inversely to the utilisation of RBM tools among these women. These variables did not influence both the acceptance and utilisation of pregnant women in faith clinics.

*Three Variables*. Number of pregnancies, educational qualification of the pregnant women, and marital status which were observed to significantly and consistently influence the acceptance and utilisation of RBM tools in this study seem to be contiguously related to one another and portend strong indications that the observed finding about these women has grave implications for their purchasing powers. Precisely, decisions about spending money are predominantly and traditionally made by men in many African cultures [24]. Also, a large number of the pregnant women who participated in the study were housewives who were with no adequate means of livelihood of their own and who had to depend on their husbands' incomes to be able to purchase the nets and the drugs. Some of the women may, therefore, not be able to make independent financial decisions since they do not have much income of their own and as such, their acceptance and utilisation of these tools could have been influenced not only by the number of pregnancies had, but also by marital status and husbands' occupations. Thus, the spouse and the nature of occupation are central to the women's responses to these tools. Findings also corroborate Pulford, Herzel, Bryant, Siba, and Mueller in [13] who reported that lack of affordability is an important barrier to ownership of ITNs. With this kind of findings, the attainment of the two main objectives of RBM programme which are “reducing the burden of malaria particularly in the two most vulnerable groups” (pregnant women and children under 5 years of age) and “contributing to the positive health and socio-economic development of the nation” is feasible and possible, all things being equal. Among the pregnant women who accessed faith clinics, the findings indicated that all the nine variables did not predict their acceptance and utilisation of the RBM tools primarily because these women are not necessarily inclined to accept or utilise these tools due to their religious beliefs and practices.

## 6. Conclusion

The study established the current status of the acceptance and utilisation of RBM programme tools (ITNs and SP in pregnancy) among pregnant women who accessed two models of healthcare facilities in Oyo State from 2001 to 2009, an initiative implemented with the purpose of stemming of malaria-related morbidity and mortality among these pregnant women and their unborn children. Though the acceptance and utilisation of RBM tools were higher among pregnant women who accessed government PHC clinics and in rural areas than among pregnant women who accessed faith clinics and in urban areas, these were far from meeting the set targets. Pregnant women's marital status and husbands' occupations significantly and statistically influenced acceptance while husbands' occupations influenced the utilisation of RBM tools implying that spouses' statuses play significant roles in achieving set targets.

We recommend that intensive effort should be made by healthcare providers to encourage especially more of the educated urban women to utilise RBM programme tools, especially the ITNs. RBM programme should not focus only on the utilisation of RBM tools but the scope should be expanded to include information dissemination on behavioural change towards malaria and its consequences. Malaria consumables such as Insecticide-Treated Nets (ITNs) and medications such as Sulphadoxine-Pyrimethamine should be made available at the various health centres and given freely. They should also be available at the open chemist stores at cheap and affordable prices to the average person.

## Figures and Tables

**Table 1:** Acceptance of IPT and ITNs by pregnant women in Oyo State.

S/N	Statement	Government clinics (*N* = 582)			Faith clinic (*N* = 50)		
		Yes %	No %	No response %	Yes %	No %	No response %
1	I like to sleep under mosquito nets.	424 (72.0)	126 (21.0)	32 (5.6)	32 (64.0)	18 (36.0)	—

2	During this pregnancy I have been sleeping under an ITN.	290 (49.8)	264 (45.4)	28 (4.8)	14 (28.0)	36 (72.0)	—

3	I feel comfortable under the ITN.	315 (54.1)	261 (44.8)	6 (1.0)	36 (72.0)	8 16.0	6 (12.0)

4	I have been coming to the clinic to receive anti-malarial drug (IPT) in this pregnancy.	304 (52.2)	232 (39.9)	46 (7.9)	15 (30.0)	27 (54.0)	8 (16.0)

5	I react to the anti-malarial drug so I do not receive it.	402 (69.1)	167 (28.7)	13 (2.2)	32 (64.0)	11 (22.0)	7 (14.0)

6	I will accept IPT in subsequent pregnancies.	390 (67.0)	170 (29.2)	22 (3.8)	31 (62.0)	14 (28.0)	5 (10.0)

**Table 2:** Summary of *t*-test analysis on acceptance of IPT and ITNs by pregnant women in terms of health facility accessed (government and faith clinics).

Status	*N*	Mean	SD	df.	*t*	*d*	*p* value
IPT in government clinics	582	4.74	1.08	630	3.51	0.14	0.000^*∗*^
IPT in faith clinics	50	4.16	1.59				

ITNs in government clinics	582	4.66	1.15	630	4.15	0.16	0.000^*∗*^
ITNs in faith clinics	50	3.96	0.93				

^*∗*^Significant at *p* ≤ 0.05.

**Table 3:** *t*-test analysis on acceptance of IPT and ITNs by pregnant women in government clinics by location.

Group	Location	*N*	Mean	SD	df.	*t*	*d*	*p* value
Acceptance of IPT by pregnant women in government clinics	Urban	278	4.55	1.13	580	−4.12	0.17	0.005^*∗*^
	Rural	304	4.92	1.00				

Acceptance of ITNs by pregnant women in government clinics	Urban	278	4.63	1.13	580	−0.58	0.024	0.565^NS^
	Rural	304	4.68	1.17				

^*∗*^Significant at *p* ≤ 0.05, NS = not significant at *p* > 0.05.

**Table 4:** Utilisation of ITNs and IPT by pregnant women in Oyo State.

S/N	Statement	Government clinics (*N* = 582)			Faith clinics (*N* = 50)		
		Yes %	No %	No response %	Yes %	No %	No response %
1	I have been sleeping under an ITN before this pregnancy.	366 (62.9)	186 (32.0)	30 (5.2)	11 (22.0)	35 (70.0)	4 (8.0)

2	Sleeping under ITN is comfortable for me.	426 (73.2)	108 (18.6)	48 (8.2)	24 (48.0)	20 (40.0)	6 (12.0)

3	I sleep under ITN daily in this pregnancy.	354 (60.8)	194 (33.3)	34 (5.8)	11 (22.0)	36 (72.0)	3 (6.0)

4	I sleep under ITN all the time.	336 (57.7)	211 (36.3)	38 (6.0)	9 (18.0)	38 (76.0)	3 (6.0)

5	I use an ITN because I find it easy to buy.	339 (58.2)	195 (33.5)	48 (8.2)	30 (60.0)	16 (32.0)	4 (8.0)

6	Two doses of anti-malarial drugs for IPT were received during this pregnancy.	389 (66.8)	180 (30.9)	13 (2.2)	19 (38.0)	22 (44.0)	9 (18.0)

7	I will like to use IPT in my subsequent pregnancies.	458 (78.7)	114 (19.6)	10 (1.7)	31 (62.0)	13 (26.0)	6 (12.0)

**Table 5:** Summary of *t*-test analysis on utilisation of IPT and ITNs by pregnant women in terms of health facility accessed (government and faith clinics).

Facility accessed	*N*	Mean	SD	df.	*t*	*d*	*p* value
IPT in government clinics	582	3.42	0.80	630	5.81	0.23	0.000^*∗*^
IPT in faith clinics	50	2.70	1.20				

ITNs in government clinics	582	7.79	2.56	630	3.99	0.16	0.000^*∗*^
ITNs in faith clinics	50	6.30	2.32				

^*∗*^Significant at *p* ≤ 0.05.

**Table 6:** Summary of *t*-test analysis on utilisation of IPT and ITNs by pregnant women in government clinics by location.

Group	Location	*N*	Mean	Std. Dev.	df.	*t*	*d*	*p* value
IPT in government clinics	Urban	278	3.20	0.87	580	−6.41	0.26	0.000^*∗*^
	Rural	304	3.61	0.67				

ITNs in government clinics	Urban	278	7.98	2.07	580	1.17	0.05	0.089^NS^
	Rural	304	7.62	2.92				

^*∗*^Significant at *p* ≤ 0.05, NS = not significant at *p* > 0.05.

**Table 7:** Correlation matrix of relationship between acceptance and utilisation of RBM tools among pregnant women in Oyo State.

Government clinics				Faith clinics	
		Acceptance	Utilisation	Acceptance	Utilisation
Acceptance	Pearson Correlation	1	0.398^*∗∗*^	1	0..379^*∗∗*^
	Sig. (2-tailed)		0.000		0.007
	*N*	582	582	50	50

Utilisation	Pearson Correlation	.398^*∗∗*^	1	0.379^*∗∗*^	1
	Sig. (2-tailed)	0.000		0.000	
	*N*	582	582	50	50

^*∗∗*^ *p* ≤ 0.01.

**Table 8:** Factors influencing acceptance of RBM tools among pregnant women in government and faith clinics.

Dependent variable	*R* ^2^	*F*	Factors influencing acceptance	*β*	*t*
Acceptance of RBM tools among pregnant women in government clinics	0.301	6.32^*∗*^	Age of pregnant woman	−0.115	−2.805^*∗*^
			Educational qualification of the pregnant woman	−0.120	−2.805^*∗*^
			Number of pregnancies had	0.217	5.172^*∗*^
			Loss of pregnancy	0.036	0.598
			Cause of miscarriage	−0.068	−1.111
			Occupation of pregnant woman	0.062	1.485
			Marital status	−0.117	−2.928^*∗*^
			Religion	0.007	0.169
			Husband's occupation	−0.093	−2.212^*∗*^

Acceptance of RBM tools among pregnant women in faith clinics	0.521	0.66^NS^			

^*∗*^Significant at *p* ≤ 0.05, NS = not significant at *p* > 0.05.

**Table 9:** Factors influencing utilisation of RBM tools among pregnant women in government and faith clinics in Oyo State.

Dependent variable	*R* ^2^	*F*	Factors influencing utilisation	*β*	*t*
Utilisation of RBM tools among pregnant women in government clinics	0.232	3.607^*∗*^	Husband's occupation	−0.071	−1.644
			Marital status	−0.132	− 3.248^*∗*^
			Age of pregnant woman	−0.070	−1.686
			Educational qualification	−0.106	−2.496^*∗*^
			Number of pregnancies had	0.121	2.818^*∗*^
			Loss of pregnancy	−0.038	−0.608
			Causes of miscarriage	−0.003	−0.054
			Husband's occupation	−0.071	−1.644
			Religion	0.033	0.791

Utilisation of RBM tools among pregnant women in faith clinics	0.392	0.807^NS^			

^*∗*^Significant at *p* ≤ 0.05, NS = not significant at *p* > 0.05.

## Abbreviations Used in the Paper

RBM: Roll Back Malaria
TBAs: Traditional Birth Attendants
ACTs: Artemisinin-Based Combination Therapies
IPT: Intermittent Preventive Treatment in pregnancy
ITNs: Insecticide-Treated Nets
LLINs: Long-Lasting Insecticides Nets
PHC: Primary Healthcare Centre
U-5: Children under five years of age
LGA: Local government area
NMCP: National Malaria Control Programme
FMOH: Federal Ministry of Health of Nigeria
FANC: Focused Antenatal Care
STDs: Sexually Transmitted Diseases
HIV/AIDS: Human Immune Virus/Acquired Immune Deficiency Syndrome
TB: Tuberculosis
WHO: World Health Organization.
